# *Salmonella enteritidis* acquires phage resistance through a point mutation in *rfbD* but loses some of its environmental adaptability

**DOI:** 10.1186/s13567-024-01341-7

**Published:** 2024-07-05

**Authors:** Yukun Zeng, Ping Li, Shenglong Liu, Mangmang Shen, Yuqing Liu, Xin Zhou

**Affiliations:** 1 College of Veterinary Medicine, Institute of Comparative Medicine, Yangzhou University, Yangzhou, 225009 China; 2 Jiangsu Co-innovation Center for Prevention and Control of Important Animal Infectious Diseases and Zoonoses, Yangzhou University, Yangzhou, 225009 China; 3 Joint International Research Laboratory of Agriculture and Agri-Product Safety, the Ministry of Education of China, Yangzhou University, Yangzhou, 225009 China; 4 Institute of Animal Science and Veterinary Medicine, Shandong Academy of Agricultural Sciences, Jinan, 250100 China

**Keywords:** *Salmonella enteritidis*, *rfbD* gene, point mutation, phage resistance, environmental adaptability

## Abstract

Phage therapy holds promise as an alternative to antibiotics for combating multidrug-resistant bacteria. However, host bacteria can quickly produce progeny that are resistant to phage infection. In this study, we investigated the mechanisms of bacterial resistance to phage infection. We found that Rsm1, a mutant strain of *Salmonella enteritidis (S. enteritidis)* sm140, exhibited resistance to phage Psm140, which was originally capable of lysing its host at sm140. Whole genome sequencing analysis revealed a single nucleotide mutation at position 520 (C → T) in the *rfbD* gene of Rsm1, resulting in broken lipopolysaccharides (LPS), which is caused by the replacement of CAG coding glutamine with a stop codon TAG. The knockout of *rfbD* in the sm140Δ*rfbD* strain caused a subsequent loss of sensitivity toward phages. Furthermore, the reintroduction of *rfbD* in Rsm1 restored phage sensitivity. Moreover, polymerase chain reaction (PCR) amplification of *rfbD* in 25 resistant strains revealed a high percentage mutation rate of 64% within the *rfbD* locus. We assessed the fitness of four bacteria strains and found that the acquisition of phage resistance resulted in slower bacterial growth, faster sedimentation velocity, and increased environmental sensitivity (pH, temperature, and antibiotic sensitivity). In short, bacteria mutants lose some of their abilities while gaining resistance to phage infection, which may be a general survival strategy of bacteria against phages. This study is the first to report phage resistance caused by *rfbD* mutation, providing a new perspective for the research on phage therapy and drug-resistant mechanisms.

**Supplementary Information:**

The online version contains supplementary material available at 10.1186/s13567-024-01341-7.

## Introduction

*Salmonella enteritidis (S. enteritidis)*, a gram-negative bacterium belonging to the Family *Enterobacteriaceae*, exists widely in different natural environments [1, 2] and has more than 2600 serotypes globally that can cause foodborne salmonellosis [3, 4]. *S. enteritidis* is the most widespread serotype found in chicken meat and is a common cause of human illnesses [5]. Antibiotics are generally used to treat bacterial infections. However, the prolonged overuse of antibiotics has led to the widespread emergence of multidrug-resistant strains of *S. enteritidis*, which in turn poses a serious threat to human health [6–10]. Over the last two decades, the inadequate supply of new antibiotics has been unable to keep up with the relentless surge of bacterial resistance. Therefore, there is an urgent need to develop new, efficient, and environmentally friendly antibacterial agents.

Bacteriophages (phages), which are estimated at 10^31^, approximately outnumbering bacteria by 100-fold, are the most abundant and diverse entities on Earth [11, 12]. Phages are widely distributed in the environment, occupying not only freshwater and marine habitats [13], surface soils [14, 15], and food sources, but also the gastrointestinal tracts of both humans and animals [16]. Phages have many advantages compared to antibiotics, antimicrobial peptides, and lysozymes. First, phages exhibit host specificity toward certain classes or species of bacteria. Second, they can be produced in large quantities and cheaply by reinfecting their bacterial hosts. Third, the phage genome demonstrates a certain degree of flexibility and can be modified through genetic engineering.

In recent years, a growing body of literature has documented the success of phage usage in the treatment of bacterial infection [17]. However, bacteria can use a range of defense strategies to fight phage infection. These strategies include modifying receptors to block phage adsorption, blocking the entry of phage DNA [18], degrading the phage genome through restriction-modification (RM) [19] and CRISPR-Cas systems [20], and preventing phage proliferation through abortive infection systems [21]. The phage receptors include various bacterial cell surface appendages, such as lipopolysaccharides (LPS), wall alginic acid, flagella, and certain, outer membrane proteins. Bacteria prevent phage adsorption and infection by shielding or blocking synthesis, or by transforming phage enzyme-mediated serotypes [22–28]. While this strategy effectively protects bacteria from phage infection, this comes at a cost to the bacteria, as the receptors involved, such as chemoreceptors, porins, and adhesins also play critical roles in bacterial metabolism and immune evasion [29].

In the process of making *S. enteritidis* resistant to phage infection, the bacterial regulation of LPS synthesis on its surface is a common resistance mechanism. The *rfbD* gene in *S. enteritidis* encodes dTDP-4-dehydrorhamnose reductase, which plays a crucial role in the synthesis of bacterial LPS [30–33]. In Gram-negative bacteria, the *rfb* region houses the genes necessary for the production and assembly of sugars, forming the side-chain repeat units of the O-antigen [34–38]. O-antigens are located in the outermost layer of LPS, in the outer membrane of bacteria [39], and play a crucial role in bacterial immune response and pathogenicity. *rfbD* catalyses the reduction of dTDP-4-dehydrOβ-l-rhamnose to dTDP-β-l-rhamnose, thereby providing essential precursors for O-antigen and lipid A synthesis. Additionally, dTDP-rhamnose plays a crucial role in LPS synthesis, ultimately forming the repeating O-antigen structure. The genetic product encoded by *rfbD* facilitates the transfer of the O-antigen from the cytoplasmic membrane to the periplasmic side, resulting in complete LPS assembly [40–42].

In this study, we isolated a mutated strain of *S. enteritidis*, Rsm1, which exhibited resistance to phages. We conducted whole genome sequencing and bioinformatic analysis to identify the specific genes responsible for facilitating this resistance. The sequencing results revealed that a single base substitution in *rfbD* caused structural damage in the LPS, which we believe is likely the cause of phage resistance in Rsm1. Our study showed for the first time that *rfbD* mutations facilitate bacterial resistance to phage infection.

## Materials and methods

### Bacterial strains, plasmids, and phages

Phage Psm140 and its host *S. enteritidis* sm140 were previously isolated from sewage and a chicken farm in Shandong Province, China, respectively, and are currently maintained in our laboratory. *S. enteritidis* sm140 belongs to the ST11 type, with a genome consisting of one chromosome (4 679 795 bp with a G + C content of 52.18%, GenBank: CP125220.1) and two plasmids (plasmid1: 64 327 bp with a G + C content of 51.76%, GenBank: CP125221.1; plasmid2: 29 336 bp with a G + C content of 47.22%, GenBank: CP125222.1). Psm140 (GenBank: PP437543) possesses a double-stranded linear DNA genome comprising 41 913 bp with a G + C content of 49.50% and belongs to the family *Siphoviridae*. Associated Professor Xiaoping Wu (College of Animal Science, Fujian Agricultural and Forestry University) provided the plasmids pCas (kanamycin-resistant) and pTarget (spectinomycin-resistant). Prof. Huoying Shi (College of Veterinary Medicine, Institute of Comparative Medicine, Yangzhou University) provided the plasmids pYA3334 (chloramphenicol-resistant) and pYA3334-Red (chloramphenicol-resistant).

### Screening and identification of phage-resistant strains

A secondary infection experiment was conducted to screen for phage-resistant strains. Fifty μL of *S. enteritidis* sm140 was inoculated in 5 mL of a lysogeny broth (LB) liquid medium and cultured until it reached an optical density at 600 nm (OD_600_) of 0.6. Phage Psm140 was then added to the culture at a multiplicity of infection (MOI) of 1:1000 and cultured overnight. Following this, the culture was streaked out, and five distinct single colonies, designated as Rsm1 to Rsm5, were selected and purified across three successive rounds without the presence of phage Psm140. The double-layer agar plate method was applied to determine the development of resistance against phage Psm140. For the strains that did not exhibit any plaques, their specificity towards *S. enteritidis* was further confirmed using the Sdf I primer (Table 1), ensuring that there was no contamination from other microorganisms.

**Table 1:** **Primers used in the experiments**

Primers	Sequences (5′-3′)	Sizes
*sdf I*	F:5′-TGTGTTTTATCTGATGCAAGAGG-3′	293 bp
	R:5′-CGTTCTTCTGGTACTTACGATGA-3′	
*rfbD*	F:5′-ATGAATATCTTACTTTTTGGTAAGACAGGG-3′	900 bp
	R:5′-TCAGATGGTTGTCGTCGTAAACATTTC-3′	
pCas	F:5′-GATACCGTCCGTTCTTTCCTT-3′	888 bp
	R:5′-TGATGATACCGCTGCCTTACT-3′	
A	F:5′-ACCGCTGGAGACCTTTGAAAG-3′	150 bp
	R:5′-GATGGGCATTTAAATTTATACTGGCGTCCTTCATAG-3′	
B	F:5′-CTATGAAGGACGCCAGTATAAATTTAAATGCCCATC-3′	153 bp
	R:5′-GTAGCAATTGCTTACTTACCGCCA-3′	
sgRNA-CRISPR	F:5′-GTCCTAGGTATAATACTAGTTtgccgggggaaccacaaccGTTTTAGAGCTAGAAATAGC-3′	2137 bp
	R:5′-ACTAGTATTATACCTAGGACTGAG-3′	
3334-*rfbD*	F:5′-CACACAGGAAACAGACCATGAATATCTTACTTTTTGGTAAGACAGG-3′	948 bp
	R:5′-GATCCCCGGGAATTGCGAATTTCAGATGGTTGTCGTCGTAAACATTTCAG-3′	
pYA3334	F:5′-AATTCGCAATTCCCGGGGATCCGTC-3′	3012 bp
	R:5′-CATGGTCTGTTTCCTGTGTGAAATTG-3′	

The lowercase sequence indicates sgRNA.

### Genome sequencing of Rsm1

The genomic DNA of Rsm1 was extracted using the TIANamp Bacteria DNA Kit (Tiangen, Beijing, China) and then submitted to Personal Biotechnology Co., Ltd. (Shanghai, China) for library construction and sequencing. The genomes of sm140 and Rsm1 were aligned using the BWA-MEM software [43], with default alignment parameters set for BWA-MEM.

The complete sequences of *rfbD* from sm140 and Rsm1 were separately amplified using the gene-specific primers *rfbD* F/R (Table 1). The amplified products were sent to TSINGKE Biological Technology Co., Ltd. (Nanjing, China) for sequencing. The subsequent analysis was conducted using MegAlign software (DNASTAR v.10.0.1).

### Validation of the phage receptor by CRISPR

The *rfbD* sequence was replicated on the CRISPOR prediction website for comprehensive analysis. A highly ranked sgRNA sequence (tgccgggggaaccacaacc) was meticulously selected. The 20 bp sgRNA fragment was then precisely cloned and inserted into the pTarget vector by PCR, using the sgRNA-CRISPR-F/R primers. Based on the sm140 genome sequence, the PCR primers AF/R and BF/R for *rfbD* were designed using the Primer Premier 5.0 software. Subsequently, the fused fragment AB was then skillfully generated by the process of overlapping PCR. Through the electroporation process, sm140 cells harboring the pCas plasmid and sm140-pCas cells harboring the pTarget-gRNA plasmid were obtained. Single clones were selected for PCR verification. The primers used in the procedures are listed in Table 1.

### *rfbD* complementation

Homologous recombination primers 3334-*rfbD* F/R for *rfbD* and pYA3334 F/R for plasmid pYA3334 (Table 1) were designed using Primer Premier 5.0 software. The genome of sm140 and the plasmid pYA3334 were used as templates to PCR-amplify the respective 3334-*rfbD* and pYA3334 fragments, employing the aforementioned primer pairs. These PCR-generated fragments were subsequently purified and seamlessly recombined via a homologous recombinase to construct the recombinant plasmid, pYA3334-*rfbD*. They were then electroporated into Rsm1 recipient cells to enable normal expression of the *rfbD* protein. Phage Psm140 was employed to assess plaque formation in both the knockout strain sm140∆*rfbD* and the complemented strain Rsm1-pYA3334-*rfbD*.

### Determination of the adsorption capacity of phage Psm140

Bacteria strains (sm140, Rsm1, sm140∆*rfbD* and Rsm1-pYA3334-*rfbD*) were cultured until reaching OD_600_ = 0.6. Subsequently, 200 μL of the bacterial suspension was mixed with 100 μL of a phage solution at a titer of 1 × 10^10^ PFU/mL (MOI = 100). 200 μL of fresh liquid LB medium was added to this mixture. As a control, 100 μL of phage solution was mixed with 400 μL of LB medium. The samples were then incubated at 37 °C for 7 min, followed by centrifugation at 12 000 ×*g* for 1 min to collect the supernatant containing the unabsorbed phages. The titer of the phages in the supernatant was an indicator of their non-adsorbed fraction. Each experiment was performed in triplicate. The bacterial pellet was resuspended in 1 × PBS and examined using TEM to determine phage-bacteria adsorption.

### Morphological characterization of the bacteria

The sm140, Rsm1, sm140∆*rfbD*, and the Rsm1-pYA3334-*rfbD* strains were individually inoculated into a liquid LB medium and cultured at 37 °C at 220 rpm until reaching an OD600 of 0.6. Subsequently, 3 μL of each culture was spotted onto solid LB agar plates and then air-dried. The plates were subsequently incubated at 37 °C for 72 h to observe the morphological differences between the bacteria strains. In addition, 10 μL of each bacteria suspension was dropped onto a copper grid and incubated at 25 °C for 10 min. An equal volume of negative staining solution containing 2% phosphotungstic acid (PTA) at pH = 7.0 was then added. The grids were air-dried. Finally, the dried grids were examined under a transmission electron microscope (TEM).

### Determination of the structural integrity of bacterial LPS

The four strains were prepared as competent cells; 100 μL of each competent cell suspension was mixed with 100 ng of the pYA3334-red plasmid. Following electroporation, the cells were incubated in antibiotic-free LB medium that had been pre-warmed to 37 °C for 1 h. Subsequently, 100 μL of the bacterial culture was plated on chloramphenicol-resistant solid LB agar. The expression of red fluorescent protein was visualized using a fluorescence microscope. Additionally, each bacterium strain containing pYA3334-Red was collected and centrifuged at 6000 × *g* for 5 min to discard the supernatant. The resulting bacterial pellets were resuspended in a solution containing 4% NaCl at 25 °C for 5 min.

The LPS of the four strains was also extracted by the hot phenol-water method [44], and SDS-PAGE was carried out, followed by silver staining.

### Detection of the mutation rate of *rfbD* in phage-resistant strains

To determine the mutation rate of *rfbD* in phage-resistant strains, additional phage-resistant mutants were screened from double-layer plates containing phage (MOI = 10) using the method described by Habusha et al. [45]. Once the bacteria clones were visible to the naked eye, 25 of these clones were randomly selected for purification and cultivation. The phage spotting method was then used to verify their resistance to phage Psm140. The *rfbD* of the phage-resistant strains was amplified and sequenced, and its mutation rate was analysed. The mutation rates were calculated using the following formula: mutation rates = (total number of mutants/total number of strains) × 100%.

### Determination of bacterial fitness before and after *rfbD* mutation

To compare the bacteria growth, nine tubes were set as follows: (1) 100 µL Rsm1 + 1.1 mL LB; (2) 100 µL Rsm1 + 1.0 mL LB + 100 µL Psm140; (3) 100 µL sm140∆*rfbD* + 1.1 mL LB; (4) 100 µL sm140∆*rfbD* + 1.0 mL LB + 100 µL Psm140; (5) 100 µL Rsm1-pYA3334-*rfbD* + 1.1 mL LB; (6) 100 µL Rsm1-pYA3334-*rfbD* + 1.0 mL LB + 100 µL Psm140; (7) 100 µL sm140 + 1.1 mL LB; (8) 100 µL sm140 + 1.0 mL LB + 100 µL Psm140; (9) 1.2 mL LB. All tubes were incubated at 37 °C, 220 rpm for 12 h to determine OD_600_. Each experiment was replicated 3 times.

To test bacteria sedimentation, 60 µL of each strain was transferred to 6 mL of liquid LB medium and incubated for 12 h. Cultures were then allowed to settle at 25 °C for 48 h to observe bacterial sedimentation.

The pH of the LB medium was adjusted using either concentrated hydrochloric acid (HCl) or sodium hydroxide (NaOH) solutions to achieve values ranging from pH = 1.0 to pH = 12.0. The four strains were separately inoculated into a liquid LB medium and were incubated at 37 °C, 220 rpm for 12 h to determine the OD600. Each experiment was replicated 3 times.

For heat treatment, the four strains were individually exposed to temperatures ranging from 37 to 80 °C for 2 h. Subsequently, 100 μL of each bacterial suspension was serially diluted and spread evenly over LB agar plates. The plates were then incubated at 37 °C for 12 h, after which the number of bacterial colonies was counted. Each experiment was replicated 3 times.

Antibiotic susceptibility testing of the four bacterial strains was performed using the Kirby-Bauer disc diffusion method in accordance with guidelines provided by the World Health Organization (WHO) [46] and the Clinical and Laboratory Standards Institute (CLSI) [47]. Bacterial suspensions were evenly spread on Mueller–Hinton agar (MHA) plates (AOBOX Biotechnology Co., Ltd. Beijing, China) and then incubated at 25 °C for 5 min. Subsequently, antibiotic discs (Hangzhou Microbial Reagent Co., Ltd. Hangzhou, China) were placed on the agar surface, and the plates were incubated at 37 °C for 16–18 h to observe the diameter of the inhibition zones. Detailed information about the antibiotics used can be found in Additional file 1.

## Results

### Screening and identification of phage-resistant strains

**Figure 1:**
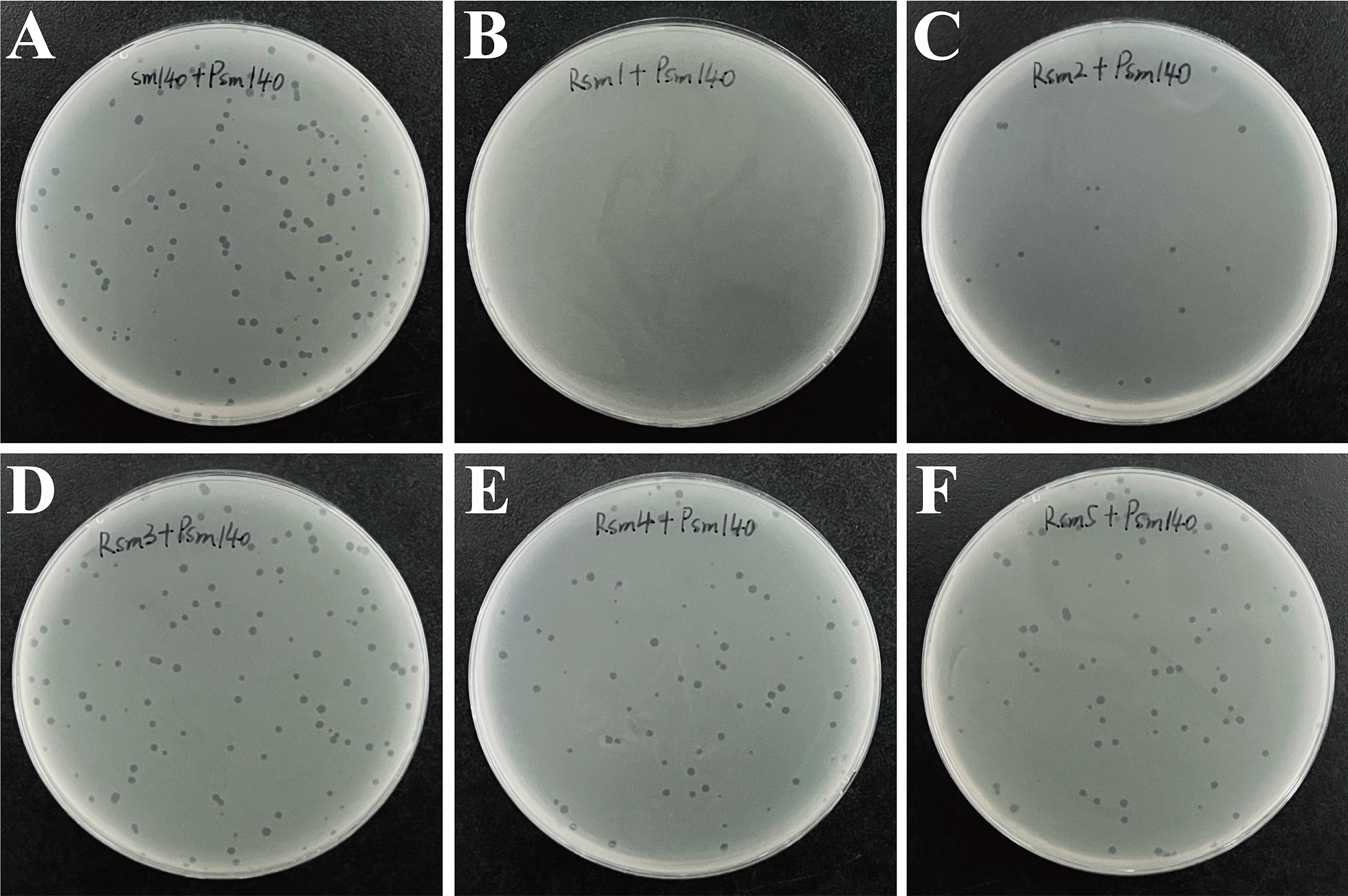
**Screening for phage-resistant strains.** **A**–**F** Results of double layer plate assays to determine the sensitivity of sm140 and Rsm1-Rsm5 to phage Psm140.

Phage resistance tests revealed that the positive control strain, sm140, was lysed by Psm140 (Figure 1A). Rsm1 exhibited resistance to Psm140 (Figure 1B). In contrast, strains Rsm2 to Rsm5 were still lysed by Psm140 (Figures 1C–F). Amplification of Rsm1, using the specific primer Sdf I for *S. enteritidis,* was followed by sequencing. Blast analysis confirmed that Rsm1 belonged to *S. enteritidis* and was not contaminated by other bacteria. Therefore, Rsm1 was selected for further experiments.

### Comparative genomic analysis and identification of Rsm1 and sm140

Rsm1 sequencing generated a dataset comprising 1144 Mbp with 7,508,608 high-quality reads. These reads were aligned to the sm140 reference genome using BWA-MEM, achieving a mapping rate of 99.81%. An analysis of single nucleotide variations using GATK software identified a single homozygous SNP within the Rsm1 genome. The raw sequencing data have been deposited online in the SRA at the NCBI, under accession number SRP475041.

**Figure 2:**
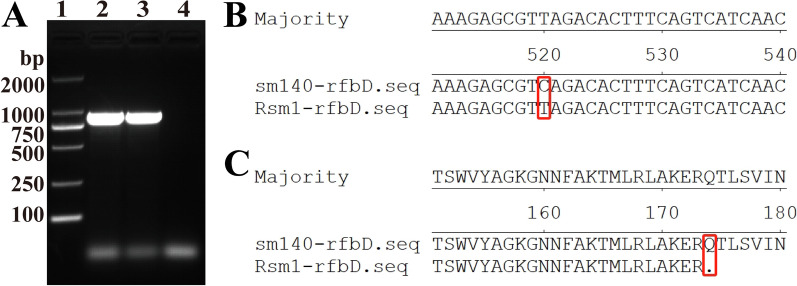
**Validation analysis of *rfbD* mutation in Rsm1.** **A** Results of *rfbD* amplification of Rsm1. Lane 1: 2000 DNA marker; Lane 2: Positive control (sm140); Lane 3: Rsm1; Lane 4: Negative control. **B**, **C** Nucleotide and amino acid alignment results of sm140 and Rsm1.

To validate the accuracy of the resequencing, PCR amplification of both the Rsm1 and sm140 *rfbD* was performed (Figure 2A), followed by sequencing alignment. This analysis confirmed a C to T mutation at position 520 of *rfbD* (Figure 2B), resulting in an amino acid substitution from glutamine to a stop codon at position 174 (Figure 2C). Consequently, the translation of *rfbD* was prematurely terminated. It was hypothesized that the abnormal expression of *rfbD* may underlie Rsm1’s tolerance to phages. To further investigate this hypothesis, we generated a *rfbD* knockout mutant for functional validation.

### *rfbD* knockout and complementation

**Figure 3:**
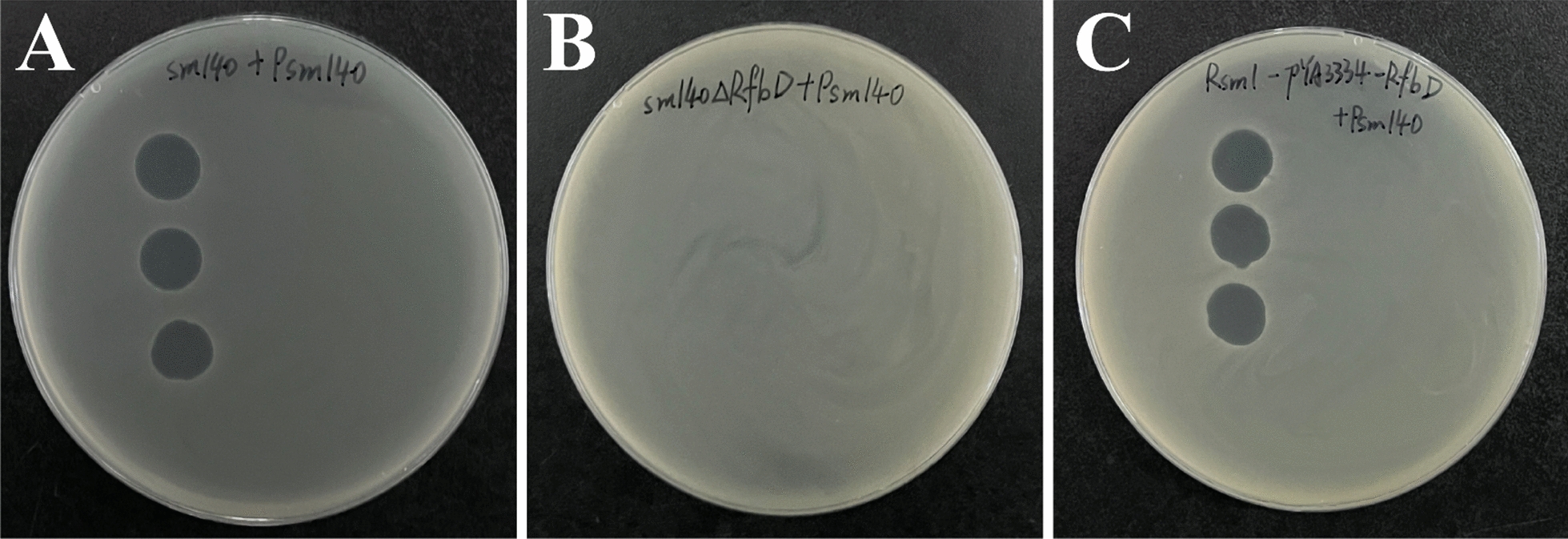
**Identification of phage sensitivity.** **A**–**C** Phage plaque assay results for sm140, sm140∆*rfbD*, and Rsm1-pYA3334-*rfbD*, respectively.

The *rfbD* knockout strain sm140∆*rfbD* was engineered using a combination of CRISPR and λ-Red technologies. The *rfbD* gene was successfully introduced into Rsm1 (Rsm1-pYA3334-*rfbD*) by employing homologous recombination technology for complementation. For the phage Psm140 plaque assay, Sm140 was used as a positive control (Figure 3A). The results indicated that sm140∆*rfbD* could not produce plaques (Figure 3B), while Rsm1-pYA3334-*rfbD* promoted plaque formation (Figure 3C). In summary, the *rfbD* knockout effectively rendered the bacteria resistant to phage-induced lysis.

### Adsorption rate of phage Psm140

**Figure 4:**
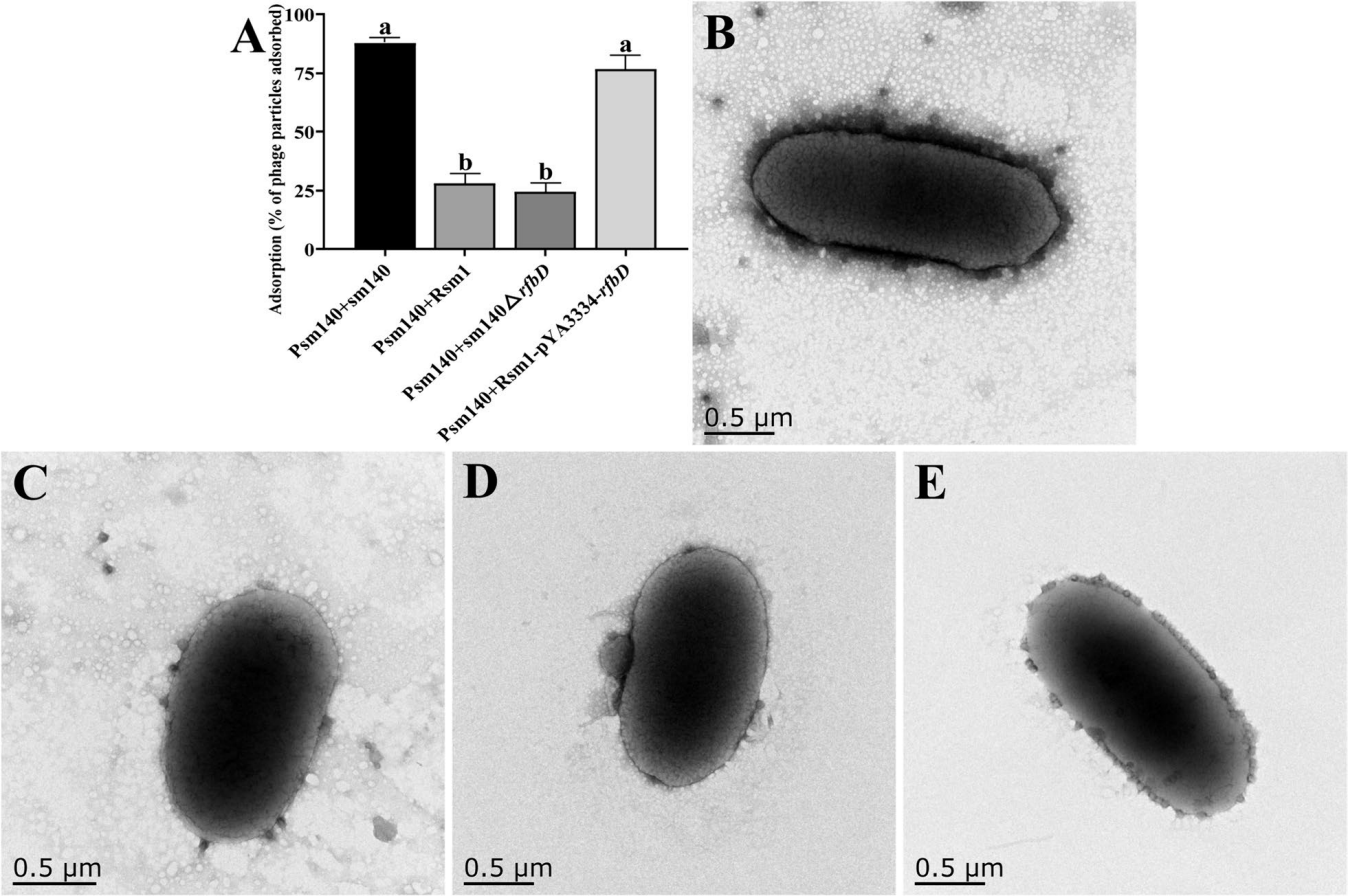
**Results of the phage adsorption test.** **A** Phage adsorption rate on bacteria. The variances among groups are delineated by comparative letter markers. Any two groups that exhibit no identical lowercase letters are considered significantly differences (*P* < 0.0001). **B**–**E** TEM images of sm140, Rsm1, sm140∆*rfbD* and Rsm1-pYA3334-*rfbD* after co-incubation with the phage for 7 min.

The adsorption rates of phage Psm140 on sm140, Rsm1, sm140∆*rfbD* and Rsm1-pYA3334-*rfbD* were 87.95, 28.22, 24.43, and 76.55%, respectively (Figure 4A). TEM revealed that the bacteria cell membrane of the wild-type strain sm140 was covered with dense phage particles (Figure 4B). In contrast, the naturally mutated Rsm1 developed resistance to phage Psm140, resulting in a significant decrease in adsorption (~59.73%), and very few phages were observed surrounding the bacteria cell, as shown in Figure 4C. These trends were also observed in the knockout strain sm140∆*rfbD* (Figure 4D). The construction of the complemented strain, Rsm1-pYA3334-*rfbD,* resulted in the phage Psm140 adsorption rate returning to its natural level. This led to the appearance of a large number of phage particles on the bacterial surface (Figure 4E).

### Analysis of bacterial morphological structure

**Figure 5:**
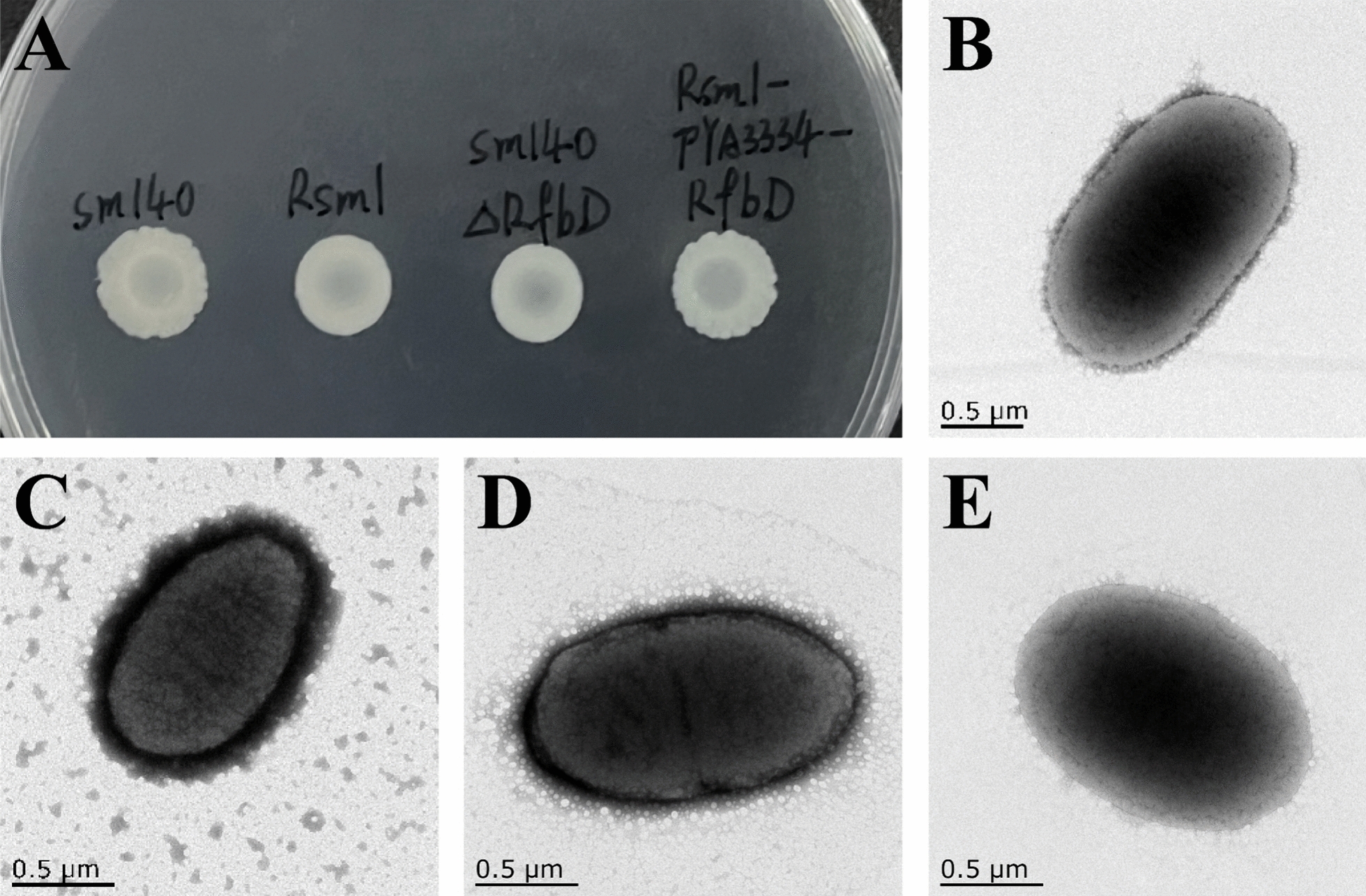
**Bacterial colony morphology and TEM characterization of bacterial morphology.** **A** Colony morphology of bacteria before and after mutation, from left to right: sm140, Rsm1, sm140∆*rfbD*, and Rsm1-pYA3334-*rfbD*. **B**–**E** TEM images of sm140, Rsm1, sm140∆*rfbD*, and Rsm1-pYA3334-*rfbD*.

Both the sm140 and Rsm1-pYA3334-*rfbD* colonies exhibited white, rough-edged appearances, in contrast to the smooth and well-defined colonies of Rsm1 and sm140∆*rfbD* (Figure 5A). TEM analysis revealed that sm140 (Figure 5B), Rsm1 (Figure 5C), sm140∆*rfbD* (Figure 5D), and Rsm1-pYA3334-*rfbD* (Figure 5E) all retained their short ellipsoidal rod shapes, measuring approximately 1.5 μm by 0.8 μm, with no significant alterations observed. The surfaces of sm140 and Rsm1-pYA3334-*rfbD* were smooth, whereas the Rsm1 and sm140∆*rfbD* surfaces displayed wrinkling, accompanied by the presence of dark dye residues.

### Determination of the integrity of bacterial LPS

**Figure 6:**
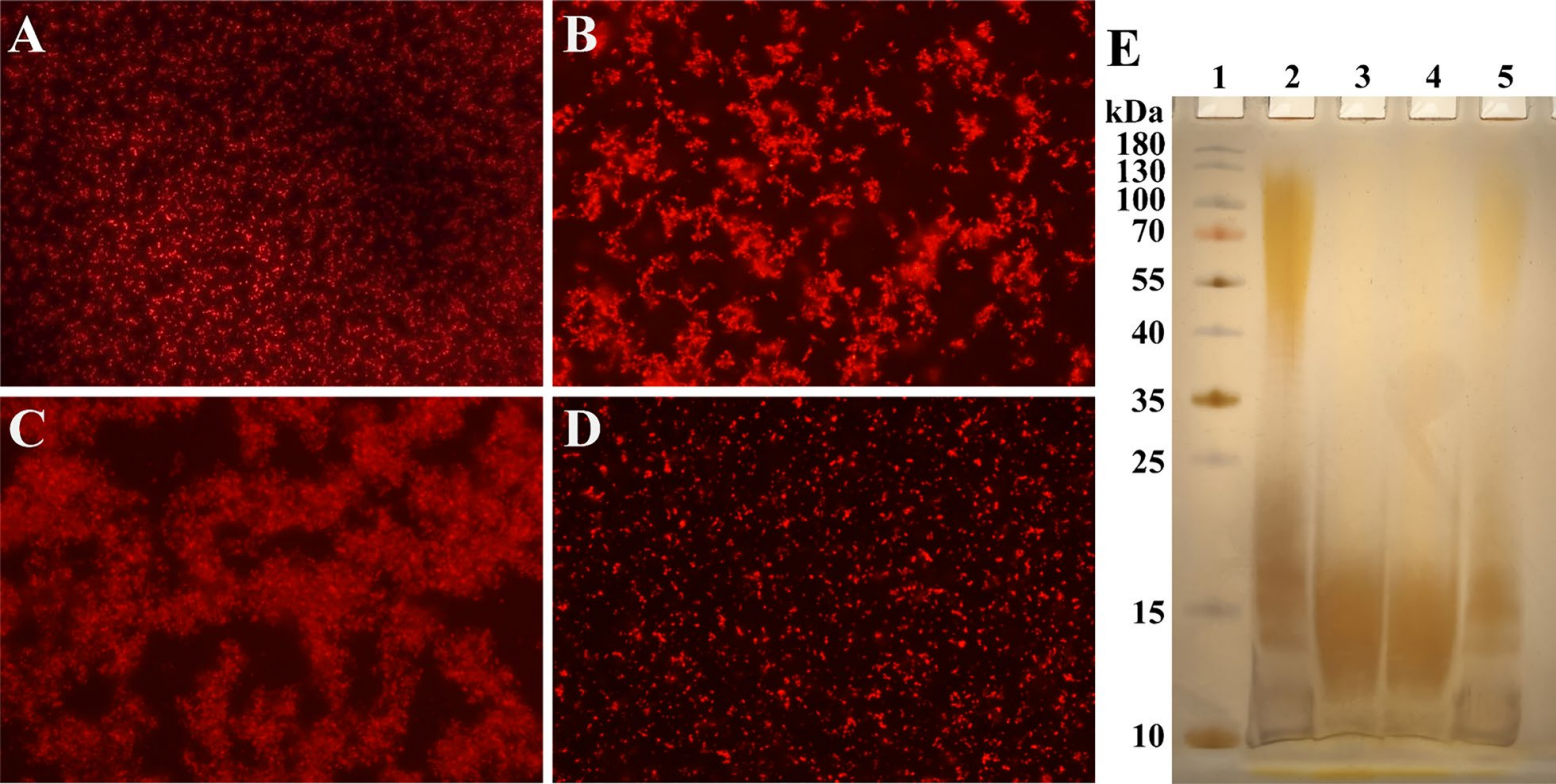
**Bacterial LPS integrity.** **A**–**D** Microscopy images showing the dispersion of sm140, Rsm1, sm140∆*rfbD*, and Rsm1-pYA3334-*rfbD* bacteria after treatment with a 4% NaCl solution. Images were taken using a fluorescence microscope at 10 × 20 magnification. **E** Silver staining of LPS. Lane 1: protein Ladder (10 ~ 180 kDa); Lane 2: sm140; Lane 3: Rsm1; Lane 4: sm140∆*rfbD*; Lane 5: Rsm1-pYA3334-*rfbD*.

The agglutination assay, a traditional method for evaluating the integrity of bacterial LPS, relies on the behavior of LPS in response to its core polysaccharide’s negative charge. In Gram-negative bacteria, this negative charge is exposed following the loss of side-chain polysaccharides on the LPS outer surface. In high-salt solutions, this, in turn, leads to aggregation. Conversely, bacteria with intact LPS structures do not aggregate and remain dispersed as individual entities, even in high-salt environments. To enhance visualization, we introduced a red fluorescent plasmid, pYA3334-red, into four strains using electroporation. The agglutination assays revealed that in a 4% NaCl solution, sm140 and Rsm1-pYA3334-*rfbD* predominantly remained individual entities, whereas Rsm1 showed partial aggregation, and sm140∆*rfbD* extensively formed aggregated patches. Subsequent silver staining experiments confirmed these findings, visibly indicating that the LPS structures of sm140 and Rsm1-pYA3334-*rfbD* were still intact. In contrast, the LPS structures of Rsm1 and sm140Δ*rfbD* were compromised, as demonstrated by their incomplete appearance, consistent with the aggregation observed in the agglutination assays (Figure 6).

### Analysis of the mutation rate of *rfbD* in phage-resistant strains

After 24 h of co-cultivating the wild-type strain sm140 with phage Psm140 on a double-layer agar plate, 138 clones of various sizes grew on the plate. The 25 randomly selected colonies were purified 3 times and designated Rsm6-Rsm30. Verification was carried out using a phage Psm140 point-plate assay, which confirmed the resistance of Rsm6-Rsm30 against phage. The sm140 was used as a control in this study. Furthermore, the *rfbD* of Rsm6-Rsm30 was amplified and sequenced, and the sequencing data were subjected to comparative analysis (Additional file 2). Among the 25 resistant strains, 64% (16/25) exhibited base replacements and frameshift mutations in *rfbD*. The remaining 36% (9/25) of the resistant strains did not show any mutation in *rfbD*. Out of these 16 mutant strains, 11 exhibited frameshift mutations due to the deletion of the fifth base. This caused the premature termination of peptide chain elongation with a mutation at the 12th amino acid into a terminator codon. Other mutations observed included a G to A substitution at position 856 (one strain), a T to G substitution at position 880 (eight strains), and an A deletion at position 892 (three strains). These all led to subsequent changes within the amino acid sequence. Overall, these mutations were likely to affect the function and stability of *rfbD*, in turn affecting the synthesis and modification of LPS, as well as its phage adsorption ability. Based on the mutation rate of *rfbD*, it was speculated that in addition to mutations within *rfbD*, sm140 may possess other mechanisms for countering phage invasion, thereby enhancing its own survival capabilities. This diversity in resistance mechanisms could be an important strategy for the survival and proliferation of *S. enteritidis* within its natural environment.

### Comparisons of bacterial fitness before and after *rfbD* mutation

**Figure 7:**
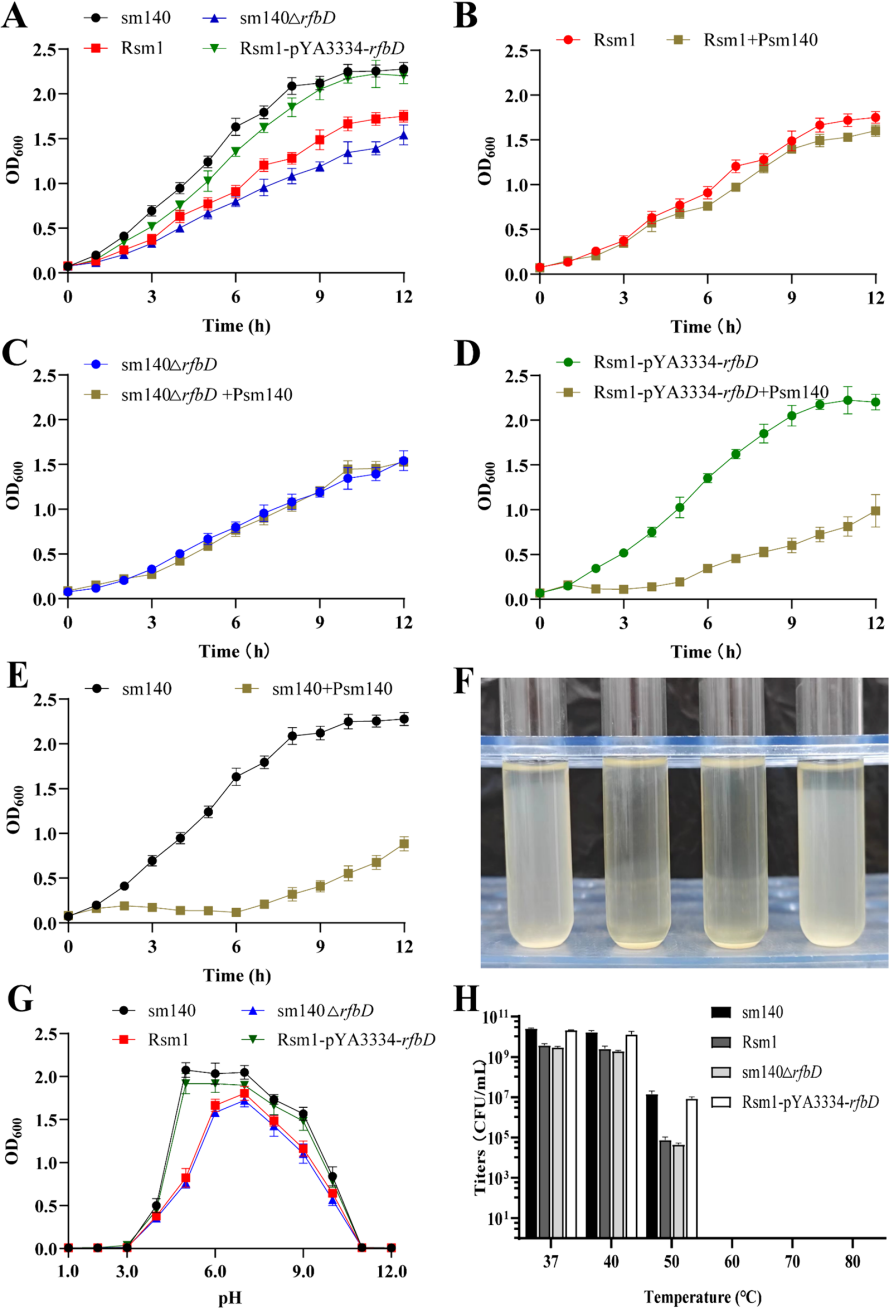
**Analysis of the physiological characteristics of the bacteria.** **A** Growth of bacteria in the absence of phage. **B**–**E** Growth of Rsm1, sm140∆*rfbD*, Rsm1-pYA3334-*rfbD*, and sm140 under co-cultivation conditions with phage Psm140. **F** Sedimentation results after 48 h of incubation at 25 °C for sm140, Rsm1, sm140∆*rfbD*, and Rsm1-pYA3334-*rfbD* (from left to right). **G**–**H** Sensitivity of bacteria to pH and temperature, respectively.

Considering that the mutation of *rfbD* conferred resistance to phage Psm140, we questioned whether this alteration affected the fitness of *S. enteritidis*. To investigate this matter further, we performed tests examining specific physiological characteristics of the bacteria, both before and after mutation. As shown in Figure 7A, the bacterial growth curve indicated that sm140’s growth significantly exceeded that of both Rsm1 and sm140Δ*rfbD*. Moreover, the growth pattern of Rsm1-pYA3334-*rfbD* closely mirrors that of sm140, with no noteworthy differences observed. Following 12 h of co-culture with Psm140 phages, as shown in Figures 7B–E, it was observed that the growth of both Rsm1 and sm140Δ*rfbD* remained largely unaffected. In contrast, significant growth inhibition was experienced by both Rsm1-pYA3334-*rfbD* and sm140.

The sedimentation velocities were assessed after 48 h at 25 °C, as shown in Figure 7F. Both sm140 and Rsm1-pYA3334-*rfbD* exhibited slower sedimentation velocities, whereas Rsm1 and sm140Δ*rfbD* strains demonstrated faster sedimentation, possibly due to self-aggregation resulting in bacterial clumping.

The optimal pH for bacterial growth was altered following mutation (Figure 7G). The optimal pH for sm140 and Rsm1-pYA3334-*rfbD* was found to be in the range of 5.0–7.0, while for Rsm1 and sm140∆*rfbD,* it remained at pH 7.0. Moreover, the temperature sensitivity of the bacteria varied after mutation (Figure 7H). At 37 and 40 °C, the growth of all four strains remained largely unaffected. However, at 50 °C, the survival rates of sm140 and Rsm1-pYA3334-*rfbD* were significantly higher than those of Rsm1 and sm140∆*rfbD*. At 60, 70, and 80 °C, the survival rates of all four bacterial strains were zero.

Antibiotic susceptibility testing revealed a significant increase in Rsm1 and sm140∆*rfbD* sensitivity to antibiotics, as detailed in Table 2. Among a total of 39 antibiotics, sm140 was resistant to 25, while Rsm1 and sm140Δ*rfbD* were resistant to only 14 and 13, respectively. These variations in drug resistance were primarily observed in antibiotics classified as β-lactams, nitrofurans, and quinolones. Following the complementation with *rfbD*, the resistance profile of Rsm1-pYA3334-*rfbD* was restored to the level of sm140. Detailed results from bacterial antibiotic susceptibility assays are provided in Additional file 1.

**Table 2:** **Drug susceptibility test results for the strains**

Strains	Number of drugs		
	Resistance (R)	Intermediate (I)	Susceptible (S)
sm140	25	2	12
Rsm1	14	8	17
sm140∆*rfbD*	13	8	18
Rsm1-pYA3334-*rfbD*	23	3	13

## Discussion

The development of phage resistance in bacteria poses a significant challenge to the effective use of phages as natural antimicrobial agents within clinical settings. Consequently, it is imperative to undertake comprehensive research into the mechanisms that help bacteria acquire phage resistance. In this study, *S. enteritidis* Rsm1 exhibited stable resistance to phage Psm140 due to mutations in *rfbD*, which is involved in the synthesis of glycosyl units, the regulation of O-antigen formation, and the integrity of LPS function. In *S. enteritidis*, the rough phenotype is typically associated with the absence of the O-antigen, leading to an irregular or uneven surface appearance. This is due to the lack of polysaccharide chains on the bacterial surface. Some specific bacterial studies have previously documented this phenomenon [34, 41, 48–50]. The presence of residual staining at the periphery of the bacterial colonies can be attributed to PTA staining’s reaction with the O-antigen on their surface. Therefore, when strains lack *rfbD* by knockout, the amount of O-antigen on the bacterial surface is reduced or is missing. This hinders the binding between PTA and bacteria and causes a black dye circle to form. On the other hand, strains with mutant *rfbD* cannot synthesize the intact O-antigen, resulting in LPS defects [51–53]. Studies have demonstrated that deleting the O-antigen glycosyltransferase gene *wadB* in *Brucella abortus* leads to an incomplete LPS structure. This leads to reduced bacterial pathogenicity and increased antibiotic sensitivity [54]. Another study showed that in *Pseudomonas extremaustralis*, the deletion of the O-antigen glycosyltransferase gene *wapH* leads to increased permeability of the bacterial outer membrane, in turn decreasing its ability to adapt to external environments [55]. In addition, mutations in the *waaC* gene, which encodes the O-antigen synthase enzyme, can affect the synthesis of O-antigen and alter the bacteria surface’s structure, hindering phage recognition and infection [56]. Similarly, mutations in the genes *rfaL* and *waaA*, which both encode ribulose synthesis, can cause abnormalities in the LPS structure, affecting the infection process of phages [57, 58]. Mutations in *RfaH* gene, which encodes a transcriptional activator and regulates LPS synthesis, can interfere with the normal generation of LPS, also resulting in bacterial resistance to phages [59].

As an initial defense against phages, bacteria typically modify or remove receptors on their surface to prevent contact with the phage [60]. However, this alteration or deletion could be costly for the host, resulting in a decrease in bacteria growth and virulence [42, 61]. In this study, mutations in *rfbD* caused a defect in LPS, making the bacteria resistant to phages and affecting their bioactivity in other ways. Similar observations have been reported for other phage-resistant strains. For example, *S. enteritidis* Salp572ɸ1R has lost O-antigen-acquired resistance to phage ɸ1, but it is almost non-virulent in mice and shows suppressed expression of certain virulence-related genes such as *cmE*, *sthE*, and *cheY* [62]. In an in vivo experiment using a calf diarrhoea model, it was found that phage B41/1 reduced the virulence of *Escherichia coli* strains [63]. Deleting the key genes *hmgA* and *galU* in *Pseudomonas aeruginosa* PA1 mutant strains resulted in the loss of O-antigen. This, in turn, led to phage resistance and reduced virulence [64]. These findings demonstrated that bacteria can develop resistance to phages by modifying or removing receptors on the surface of the bacteria. However, such changes often sacrifice bioactivity, indicating the trade-offs and challenges bacteria face regarding survival and reproduction. Achieving a balance between phage resistance and bacterial viability may be a survival strategy for bacteria. By studying the mutation principles of bacteriophage resistance, it is possible to develop universal bactericidal phage reagents for treating patients with clinical bacterial infection.

In this study, we confirmed that the resistance of Rsm1 to phage Psm140 stemmed from a point mutation in the Rsm1 genome, located at the 520 bp position in the *rfbD* gene (C → T transition). The consequent resistance of bacteria to phages, which also reduced their adaptive capabilities, was clearly demonstrated through targeted knockout experiments of *rfbD*, followed by complementation studies. This is the first report on a mutation in *rfbD* conferring phage resistance, and this finding provides a theoretical basis for studying phage therapy and resistance mechanisms in *S. enteritidis* infection.

### Supplementary Information

**Additional file 1**:** Drug susceptibility test results for the strains.**

**Additional file 2**:** Sequence alignment analysis of** ***rfbD* in phage-resistant strains.**

## Abbreviations

LPS: Lipopolysaccharides
PTA: Phosphotungstic acid
TEM: Transmission electron microscope
HCl: Hydrochloric acid
NaOH: Sodium hydroxide
CLSI: Clinical and Laboratory Standards Institute
SNP: Single nucleotide polymorphism
KDO: 3-Deoxy-D-manno-octulosonic acid
WTAs: Wall teichoic acids

## References

[1] Chiu LH, Chiu CH, Horn YM, Chiou CS, Lee CY, Yeh CM, Yu CY, Wu CP, Chang CC, Chu C (2010). Characterization of 13 multi-drug resistant *Salmonella* serovars from different broiler chickens associated with those of human isolates. BMC Microbiol.

[2] Scallan E, Hoekstra RM, Angulo FJ, Tauxe RV, Widdowson MA, Roy SL, Jones JL, Griffin PM (2011). Foodborne illness acquired in the United States–major pathogens. Emerg Infect Dis.

[3] Guibourdenche M, Roggentin P, Mikoleit M, Fields PI, Bockemühl J, Grimont PA, Weill FX (2010). Supplement 2003–2007 (No. 47) to the White-Kauffmann-Le Minor scheme. Res Microbiol.

[4] Chen S, Zhao S, White DG, Schroeder CM, Lu R, Yang H, McDermott PF, Ayers S, Meng J (2004). Characterization of multiple-antimicrobial-resistant *salmonella* serovars isolated from retail meats. Appl Environ Microbiol.

[5] Thai TH, Hirai T, Lan NT, Yamaguchi R (2012). Antibiotic resistance profiles of *Salmonella* serovars isolated from retail pork and chicken meat in North Vietnam. Int J Food Microbiol.

[6] Butaye P, Michael GB, Schwarz S, Barrett TJ, Brisabois A, White DG (2006). The clonal spread of multidrug-resistant non-typhi *Salmonella* serotypes. Microbes Infect.

[7] Klontz KC, Klontz JC, Mody RK, Hoekstra RM (2010). Analysis of tomato and jalapeño and Serrano pepper imports into the United States from Mexico before and during a national outbreak of *Salmonella* serotype Saintpaul infections in 2008. J Food Prot.

[8] McGuinness S, McCabe E, O’Regan E, Dolan A, Duffy G, Burgess C, Fanning S, Barry T, O’Grady J (2009). Development and validation of a rapid real-time PCR based method for the specific detection of *Salmonella* on fresh meat. Meat Sci.

[9] Smith SI, Fowora MA, Goodluck HA, Nwaokorie FO, Aboaba OO, Opere B (2011). Molecular typing of *Salmonella* spp isolated from food handlers and animals in Nigeria. Int J Mol Epidemiol Genet.

[10] Cabello FC (2006). Heavy use of prophylactic antibiotics in aquaculture: a growing problem for human and animal health and for the environment. Environ Microbiol.

[11] Brüssow H, Hendrix RW (2002). Phage genomics: small is beautiful. Cell.

[12] Fortier LC, Sekulovic O (2013). Importance of prophages to evolution and virulence of bacterial pathogens. Virulence.

[13] Srinivasiah S, Bhavsar J, Thapar K, Liles M, Schoenfeld T, Wommack KE (2008). Phages across the biosphere: contrasts of viruses in soil and aquatic environments. Res Microbiol.

[14] Williamson KE, Radosevich M, Wommack KE (2005). Abundance and diversity of viruses in six Delaware soils. Appl Environ Microbiol.

[15] Prigent M, Leroy M, Confalonieri F, Dutertre M, DuBow MS (2005). A diversity of bacteriophage forms and genomes can be isolated from the surface sands of the Sahara Desert. Extremophiles.

[16] Shkoporov AN, Hill C (2019). Bacteriophages of the human gut: the “known unknown” of the microbiome. Cell Host Microbe.

[17] Li GM (2008). Mechanisms and functions of DNA mismatch repair. Cell Res.

[18] Kintz E, Davies MR, Hammarlöf DL, Canals R, Hinton JC, van der Woude MW (2015). A BTP1 prophage gene present in invasive non-typhoidal *Salmonella* determines composition and length of the O-antigen of the lipopolysaccharide. Mol Microbiol.

[19] Sneppen K, Semsey S, Seshasayee AS, Krishna S (2015). Restriction modification systems as engines of diversity. Front Microbiol.

[20] Al-Shayeb B, Sachdeva R, Chen LX, Ward F, Munk P, Devoto A, Castelle CJ, Olm MR, Bouma-Gregson K, Amano Y, He C, Méheust R, Brooks B, Thomas A, Lavy A, Matheus-Carnevali P, Sun C, Goltsman DSA, Borton MA, Sharrar A, Jaffe AL, Nelson TC, Kantor R, Keren R, Lane KR, Farag IF, Lei S, Finstad K, Amundson R, Anantharaman K, Zhou J, Probst AJ, Power ME, Tringe SG, Li WJ, Wrighton K, Harrison S, Morowitz M, Relman DA, Doudna JA, Lehours AC, Warren L, Cate JHD, Santini JM, Banfield JF (2020). Clades of huge phages from across Earth’s ecosystems. Nature.

[21] Sekulovic O, Ospina Bedoya M, Fivian-Hughes AS, Fairweather NF, Fortier LC (2015). The *Clostridium difficile* cell wall protein CwpV confers phase-variable phage resistance. Mol Microbiol.

[22] Allison GE, Verma NK (2000). Serotype-converting bacteriophages and O-antigen modification in *Shigella flexneri*. Trends Microbiol.

[23] Scholl D, Adhya S, Merril C (2005). *Escherichia coli* K1’s capsule is a barrier to bacteriophage T7. Appl Environ Microbiol.

[24] Høyland-Kroghsbo NM, Maerkedahl RB, Svenningsen SL (2013). A quorum-sensing-induced bacteriophage defense mechanism. mBio.

[25] Manning AJ, Kuehn MJ (2011). Contribution of bacterial outer membrane vesicles to innate bacterial defense. BMC Microbiol.

[26] Hoque MM, Naser IB, Bari SM, Zhu J, Mekalanos JJ, Faruque SM (2016). Quorum regulated resistance of *Vibrio cholerae* against environmental bacteriophages. Sci Rep.

[27] Høyland-Kroghsbo NM, Paczkowski J, Mukherjee S, Broniewski J, Westra E, Bondy-Denomy J, Bassler BL (2017). Quorum sensing controls the *Pseudomonas aeruginosa* CRISPR-Cas adaptive immune system. Proc Natl Acad Sci U S A.

[28] Bondy-Denomy J, Qian J, Westra ER, Buckling A, Guttman DS, Davidson AR, Maxwell KL (2016). Prophages mediate defense against phage infection through diverse mechanisms. ISME J.

[29] Hyman P, Abedon ST (2010). Bacteriophage host range and bacterial resistance. Adv Appl Microbiol.

[30] Dong C, Beis K, Giraud MF, Blankenfeldt W, Allard S, Major LL, Kerr ID, Whitfield C, Naismith JH (2003). A structural perspective on the enzymes that convert dTDP-d-glucose into dTDP-l-rhamnose. Biochem Soc Trans.

[31] Giraud MF, Naismith JH (2000). The rhamnose pathway. Curr Opin Struct Biol.

[32] Law A, Stergioulis A, Halavaty AS, Minasov G, Anderson WF, Kuhn ML (2017). Structure of the *Bacillus anthracis* dTDP-L-rhamnose-biosynthetic enzyme dTDP-4-dehydrorhamnose reductase (RfbD). Acta Crystallogr F Struct Biol Commun.

[33] Vinh T, Adler B, Faine S (1986). Ultrastructure and chemical composition of lipopolysaccharide extracted from *Leptospira interrogans* serovar *copenhageni*. J Gen Microbiol.

[34] Xiang SH, Haase AM, Reeves PR (1993). Variation of the rfb gene clusters in *Salmonella enterica*. J Bacteriol.

[35] Macpherson DF, Manning PA, Morona R (1994). Characterization of the dTDP-rhamnose biosynthetic genes encoded in the rfb locus of *Shigella flexneri*. Mol Microbiol.

[36] Formal SB, Gemski P, Baron LS, Labrec EH (1970). Genetic transfer of *Shigella flexneri* antigens to *Escherichia coli* K-12. Infect Immun.

[37] Petrovskaya VG, Licheva TA (1982). A provisional chromosome map of *Shigella* and the regions related to pathogenicity. Acta Microbiol Acad Sci Hung.

[38] Whitfield C, Roberts IS (1999). Structure, assembly and regulation of expression of capsules in *Escherichia coli*. Mol Microbiol.

[39] Kim M, Kim S, Park B, Ryu S (2014). Core lipopolysaccharide-specific phage SSU5 as an auxiliary component of a phage cocktail for *Salmonella* biocontrol. Appl Environ Microbiol.

[40] Lalsiamthara J, Kaur G, Gogia N, Ali SA, Goswami TK, Chaudhuri P (2020). *Brucella abortus* S19 *rfbD* mutant is highly attenuated, DIVA enable and confers protection against virulent challenge in mice. Biologicals.

[41] Mitchison M, Bulach DM, Vinh T, Rajakumar K, Faine S, Adler B (1997). Identification and characterization of the dTDP-rhamnose biosynthesis and transfer genes of the lipopolysaccharide-related rfb locus in *Leptospira interrogans* serovar *Copenhageni*. J Bacteriol.

[42] Zhou Y, Xiong D, Guo Y, Liu Y, Kang X, Song H, Jiao X, Pan Z (2023). *Salmonella Enteritidis* RfbD enhances bacterial colonization and virulence through inhibiting autophagy. Microbiol Res.

[43] Li H, Durbin R (2009). Fast and accurate short read alignment with Burrows-Wheeler transform. Bioinformatics.

[44] Conrad RS, Galanos C (1989). Fatty acid alterations and polymyxin B binding by lipopolysaccharides from *Pseudomonas aeruginosa* adapted to polymyxin B resistance. Antimicrob Agents Chemother.

[45] Habusha M, Tzipilevich E, Fiyaksel O, Ben-Yehuda S (2019). A mutant bacteriophage evolved to infect resistant bacteria gained a broader host range. Mol Microbiol.

[46] Guzman M, Dille J, Godet S (2012). Synthesis and antibacterial activity of silver nanoparticles against gram-positive and gram-negative bacteria. Nanomedicine.

[47] Khoshbakht R, Salimi A, Shirzad Aski H, Keshavarzi H (2013). Antibiotic susceptibility of bacterial strains isolated from urinary tract infections in Karaj, Iran. Jundishapur J Microbiol.

[48] Gao M, D’Haeze W, De Rycke R, Wolucka B, Holsters M (2001). Knockout of an azorhizobial dTDP-L-rhamnose synthase affects lipopolysaccharide and extracellular polysaccharide production and disables symbiosis with *Sesbania rostrata*. Mol Plant Microbe Interact.

[49] Jayeola V, McClelland M, Porwollik S, Chu W, Farber J, Kathariou S (2020). Identification of novel genes mediating survival of *Salmonella* on low-moisture foods via transposon sequencing analysis. Front Microbiol.

[50] Robertson BD, Frosch M, van Putten JP (1994). The identification of cryptic rhamnose biosynthesis genes in *Neisseria gonorrhoeae* and their relationship to lipopolysaccharide biosynthesis. J Bacteriol.

[51] Whitfield C (2006). Biosynthesis and assembly of capsular polysaccharides in *Escherichia coli*. Annu Rev Biochem.

[52] Raetz CR, Whitfield C (2002). Lipopolysaccharide endotoxins. Annu Rev Biochem.

[53] Woodward JJ, Iavarone AT, Portnoy DA (2010). c-di-AMP secreted by intracellular *Listeria monocytogenes* activates a host type I interferon response. Science.

[54] Arce G, Iriarte H, Zuniga R, Gil R (2014). The identification of *wadB*, a new glycosyltransferase gene, confirms the branched structure and the role in virulence of the lipopolysaccharide core of *Brucella abortus*. Microb Pathog.

[55] Benforte FC, Colonnella MA, Ricardi MM, Solar VEC, Leonardo L, López Nancy I, Tribelli PM, Appanna VD (2018). Novel role of the LPS core glycosyltransferase WapH for cold adaptation in the Antarctic bacterium *Pseudomonas extremaustralis*. PLoS ONE.

[56] Valvano MA, Messner P, Kosma P (2002). Novel pathways for biosynthesis of nucleotide-activated *glycero-manno*-heptose precursors of bacterial glycoproteins and cell surface polysaccharides. Microbiology.

[57] Kneidinger B, Marolda C, Graninger M, Zamyatina A, McArthur F, Kosma P, Valvano MA, Messner P (2002). Biosynthesis pathway of ADP-L-glycero-beta-D-manno-heptose in *Escherichia coli*. J Bacteriol.

[58] Donohue-Rolfe A, Kondova I, Oswald S, Hutto D, Tzipori S (2000). *Escherichia coli* O157:H7 strains that express Shiga toxin (Stx) 2 alone are more neurotropic for gnotobiotic piglets than are isotypes producing only Stx1 or both Stx1 and Stx2. J Infect Dis.

[59] Murray GL, Attridge SR, Morona R (2003). Regulation of *Salmonella typhimurium* lipopolysaccharide O antigen chain length is required for virulence; identification of FepE as a second Wzz. Mol Microbiol.

[60] Labrie SJ, Samson JE, Moineau S (2010). Bacteriophage resistance mechanisms. Nat Rev Microbiol.

[61] Capparelli R, Nocerino N, Lanzetta R, Silipo A, Amoresano A, Giangrande C, Becker K, Blaiotta G, Evidente A, Cimmino A, Iannaccone M, Parlato M, Medaglia C, Roperto S, Roperto F, Ramunno L, Iannelli D (2010). Bacteriophage-resistant
* Staphylococcus aureus
* mutant confers broad immunity against staphylococcal infection in mice. PLoS ONE.

[62] Capparelli R, Nocerino N, Iannaccone M, Ercolini D, Parlato M, Chiara M, Iannelli D (2010). Bacteriophage therapy of *Salmonella enterica*: a fresh appraisal of bacteriophage therapy. J Infect Dis.

[63] Smith HW, Huggins MB, Shaw KM (1987). The control of experimental *Escherichia coli* diarrhoea in calves by means of bacteriophages. J Gen Microbiol.

[64] Le S, Yao X, Lu S, Tan Y, Rao X, Li M, Jin X, Wang J, Zhao Y, Wu NC, Lux R, He X, Shi W, Hu F (2014). Chromosomal DNA deletion confers phage resistance to *Pseudomonas aeruginosa*. Sci Rep.

