# Supplementary material for: Salmonella enteritidis acquires phage resistance through a point mutation in rfbD but loses some of its environmental adaptability
Source: Vet Res. 2024 Jul 5;55:85. doi: 10.1186/s13567-024-01341-7 (PMC11227202; doi:10.1186/s13567-024-01341-7)
Supplement: Supplementary file 1 — Additional file 1: Drug susceptibility test results for the strains. [file 13567_2024_1341_MOESM1_ESM.docx]

**Additional file 1 Drug susceptibility test results of strains**

| Antibiotics type | Antibiotics | Inhibition circle diameter (mm) | | | Strains | | | |
| --- | --- | --- | --- | --- | --- | --- | --- | --- |
|  |  | Resistance  (R) | Intermediate  (I) | Susceptible  (S) | sm140 | Rsm1 | sm140∆*rfbD* | Rsm1-pYA3334-*rfbD* |
| β-lactams | Penicillin G (10 μg) | ＜13 | 13-15 | ≥16 | R | R | R | R |
|  | Oxacillin (1 μg) | ≤10 | 11-12 | ≥13 | R | R | R | R |
|  | Sultamicillin (10 μg) | ≤13 | 14-16 | ≥17 | R | R | R | R |
|  | Carbenicillin (100 μg) | ≤19 | 20-22 | ≥23 | R | R | R | R |
|  | Tazocin (10 μg) | ≤17 | 18-20 | ≥21 | R | R | R | R |
|  | Cefalexin (30 μg) | ≤14 | 15-17 | ≥18 | R | I | I | R |
|  | Cefamezin (30 μg) | ≤14 | 15-17 | ≥18 | S | S | S | S |
|  | Cefradine (30 μg) | ≤14 | 15-17 | ≥18 | R | I | I | R |
|  | Cefuroxim (30 μg) | ≤14 | 15-22 | ≥23 | R | R | I | R |
|  | Ceftazidime (30 μg) | ≤14 | 15-17 | ≥18 | R | S | S | R |
|  | Ceftriaxone (30 μg) | ≤13 | 14-20 | ≥21 | S | S | S | S |
|  | Cefperazon-Sulbact (75 μg) | ≤15 | 16-20 | ≥21 | R | I | I | R |
|  | Cefepime (30 μg) | ≤14 | 15-17 | ≥18 | S | S | S | S |
|  | Cefoxitin (30 μg) | ≤14 | 15-17 | ≥18 | R | I | I | I |
|  | Cefotaxime (30 μg) | ≤14 | 15-22 | ≥23 | S | S | S | S |
|  | Imipenem (10 μg) | ≤13 | 14-15 | ≥16 | S | S | S | S |
|  | Aztreonam (30 μg) | ≤15 | 16-21 | ≥22 | S | S | S | S |
| Nitrofurans | Furadantin (300 μg) | ≤14 | 15-16 | ≥17 | I | S | S | I |
|  | Furazolidone (300 μg) | ≤14 | 15-16 | ≥17 | R | I | I | R |
| Macrolides | Azithromycin (15 μg) | ≤13 | 14-17 | ≥18 | R | R | R | R |
|  | Medemcyin (30 μg) | ≤13 | 14-17 | ≥18 | R | R | R | R |
|  | Erythromycin (15 μg) | ≤13 | 14-22 | ≥23 | R | R | R | R |
| Aminoglycosides | Gentamicin (10 μg) | ≤12 | 13-14 | ≥15 | R | I | I | R |
|  | Neomycin (30 μg) | ≤12 | 13-16 | ≥17 | R | I | I | R |
|  | Tobramycin (10 μg) | ≤12 | 13-14 | ≥15 | I | S | S | S |
|  | Kanamycin (30 μg) | ≤13 | 14-17 | ≥18 | S | S | S | S |
|  | Amikacin (30 μg) | ≤14 | 15-16 | ≥17 | S | S | S | S |
| Quinolones | Levofloxacin (5 μg) | ≤13 | 14-16 | ≥17 | R | S | S | R |
|  | Norfloxacin (10 μg) | ≤12 | 13-16 | ≥17 | S | S | S | S |
|  | Ciprofloxacin (5 μg) | ≤15 | 16-20 | ≥21 | R | I | S | I |
| Sulfonamides | Trimethoprim (5 μg) | ≤10 | 11-15 | ≥16 | R | S | S | R |
|  | Compound Sulfamethoxazole (3.75/1.25 μg) | ≤10 | 11-16 | ≥17 | S | S | S | S |
| Glycopeptides | Vancomycin (30 μg) | ≤14 | 15-16 | ≥17 | R | R | R | R |
| Polypeptides | Polymixin B (300 IU) | ≤8 | 9-11 | ≥12 | S | S | S | S |
| Tetracyclines | Tetracycline (30 μg) | ≤14 | 15-18 | ≥19 | R | R | R | R |
|  | Doxycycline (30 μg) | ≤12 | 13-15 | ≥16 | R | R | R | R |
|  | Minocycline (30 μg) | ≤14 | 15-18 | ≥19 | R | R | R | R |
| Chloromycetin | Chloramphenicol (30 μg) | ≤12 | 13-17 | ≥18 | S | S | S | S |
| Lincomycin | Clindamycin (2 μg) | ≤14 | 15-20 | ≥21 | R | R | R | R |

**Note:** Antibiotic susceptibility testing of the four bacterial strains was performed using the Kirby-Bauer disc diffusion method according to World Health Organization and the Clinical and Laboratory Standards Institute (CLSI) guidelines.
