# Supplementary material for: Salmonella enteritidis acquires phage resistance through a point mutation in rfbD but loses some of its environmental adaptability
Source: Vet Res. 2024 Jul 5;55:85. doi: 10.1186/s13567-024-01341-7 (PMC11227202; doi:10.1186/s13567-024-01341-7)
Supplement: Supplementary file 2 — Additional file 2: Sequence alignment analysis of rfbD in phage-resistant strains. [file 13567_2024_1341_MOESM2_ESM.docx]

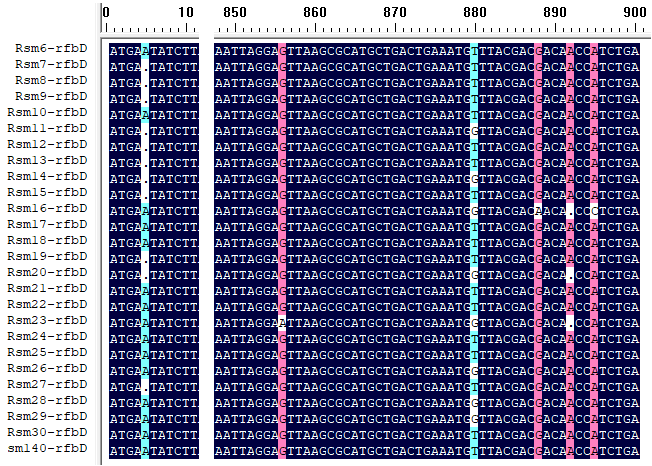


**Additional file 2 Sequence alignment analysis of *rfbD* in phage-resistant strains.** Deep blue indicates 100% homology in base composition at that position; pink indicates base homology ≥ 75%; light blue indicates base homology ≥ 50%; "." indicates a base deletion at that specific position.
